# p21^WAF1/CIP1^ RNA Expression in Highly HIV-1 Exposed, Uninfected Individuals

**DOI:** 10.1371/journal.pone.0119218

**Published:** 2015-03-06

**Authors:** Joshua Herbeck, Suvankar Ghorai, Lennie Chen, Charles R. Rinaldo, Joseph B. Margolick, Roger Detels, Lisa Jacobson, Steven Wolinsky, James I. Mullins

**Affiliations:** 1 Department of Global Health, University of Washington, Seattle, Washington, United States of America; 2 Department of Microbiology, University of Washington, Seattle, Washington, United States of America; 3 University of Pittsburgh, Pittsburgh, Pennsylvania, United States of America; 4 Department of Molecular Microbiology and Immunology, Johns Hopkins University, Baltimore, Maryland, United States of America; 5 Department of Epidemiology, University of California Los Angeles, Los Angeles, California, United States of America; 6 Department of Epidemiology, Johns Hopkins University, Baltimore, Maryland, United States of America; 7 Department of Medicine, Northwestern University, Chicago, Illinois, United States of America; University of Cape Town, SOUTH AFRICA

## Abstract

Some individuals remain HIV-1 antibody and PCR negative after repeated exposures to the virus, and are referred to as HIV-exposed seronegatives (HESN). However, the causes of resistance to HIV-1 infection in cases other than those with a homozygous CCR5Δ32 deletion are unclear. We hypothesized that human p21^WAF1/CIP1^ (a cyclin-dependent kinase inhibitor) could play a role in resistance to HIV-1 infection in HESN, as p21 expression has been associated with suppression of HIV-1 in elite controllers and reported to block HIV-1 integration in cell culture. We measured p21 RNA expression in PBMC from 40 HESN and 40 low exposure HIV-1 seroconverters (LESC) prior to their infection using a real-time PCR assay. Comparing the 20 HESN with the highest exposure risk (median = 111 partners/2.5 years prior to the 20 LESC with the lowest exposure risk (median = 1 partner/2.5 years prior), p21 expression trended higher in HESN in only one of two experiments (P = 0.11 vs. P = 0.80). Additionally, comparison of p21 expression in the top 40 HESN (median = 73 partners/year) and lowest 40 LESC (median = 2 partners/year) showed no difference between the groups (P = 0.84). There was a weak linear trend between risk of infection after exposure and increasing p21 gene expression (R^2^ = 0.02, P = 0.12), but again only in one experiment. Hence, if p21 expression contributes to the resistance to viral infection in HESN, it likely plays a minor role evident only in those with extremely high levels of exposure to HIV-1.

## Introduction

It is now well established that some persons who have been exposed to HIV-1 repeatedly, or who exhibit behaviors associated with a high risk of infection, can remain uninfected [1,2,3,4]. These individuals have been referred to as HIV-exposed seronegatives (HESN) [1], and represent a uniquely valuable group for identifying potential host factors and/or immune correlates of resistance to HIV infection.

To date, the only host genetic factor consistently found to be associated with protection from HIV infection is a homozygous 32 base pair deletion in the CCR5 gene, which encodes a major viral coreceptor [5,6,7]. It has been suggested that variations in beta chemokine ligands can result in blockade or sequestering of CCR5 and thereby also affect the relative risk or protection from infection with HIV [8,9]. Reports based on gene expression knockdown studies have suggested that several other factors, alone or in combination, may contribute to protection from HIV infection [10,11,12]. Additional candidates suggested to affect host susceptibility to HIV-1 infection include TRIM5α [13,14], specific KIR-HLA associations [15], APOBEC3G [16,17], TAP2 Ala665 [18], IRF1 [19], DC-SIGN [20], and TLR9 [21].

p21 is a well-known cyclin-dependent kinase inhibitor (CKI) that can lead to cell cycle arrest upon DNA damage [22,23,24], particularly of activated/memory T cells in mouse studies [25]. p21 has also been found to be a potent inhibitor of HIV-1 DNA integration, and to be responsible for resistance to HIV-1 infection in primitive hematopoietic cells [26], activated CD4+ T cells and monocyte-derived macrophages [27,28]. Moreover, high levels of expression of p21 have been reported to lower HIV production levels in cell culture [29]. However, others have found that although p21 expression was higher in CD4+ T cells from HIV controllers than from healthy controls, it did not correlate with CD4+ T-cell susceptibility to HIV-1 infection [30].

Based primarily on the data demonstrating suppression of viral integration and resistance to cellular infection [26,28], we reasoned that individuals with high p21 levels may exhibit resistance to HIV-1 infection. To test this hypothesis, we studied p21 RNA expression levels in HIV seronegative individuals with risk behaviors suggestive of unusually high levels of HIV exposure, and used as a comparison group HIV-uninfected individuals with reported low risk behavior but who nonetheless subsequently acquired HIV-1 infection.

## Methods

### Study group and ethics statement

The institutional review boards of MACS participating institutions (University of Pittsburgh; Johns Hopkins University; University of California, Los Angeles; Northwestern University) approved the MACS study protocol, and written informed consent was obtained from all participants. The Multicenter AIDS Cohort (MACS), a longitudinal prospective cohort study of HIV infection in the United States, enrolled 6973 men between the years 1984–2003 [31]. The MACS collects plasma and peripheral blood mononuclear cells, and clinical data, from HIV-infected and HIV-uninfected study participants at six-month intervals. The MACS defined a quantitative estimate of HIV exposure risk, the partner score (*pscore*), determined by the number of self-reported different sexual partners and the proportion of those partners with whom the individual had unprotected anal-receptive intercourse in the 2.5 years before MACS semiannual visit 2 (which took place in 1985) [32].

For the current study, we identified HESN and low exposure HIV-1 seroconverters (LESC) by ranking individuals by *pscore*. Our initial group of HESN included 20 individuals with the highest pscores and who remained seronegative for >15 years, studying only individuals of European descent (for matching purposes in this case-control study) and those that did not possess any CCR5Δ32 alleles. The initial group of LESC contained 20 HIV-infected individuals with the lowest *pscores*, and only individuals of European descent without CCR5Δ32 alleles. All samples from LESC were obtained 3–9 months prior to HIV-1 infection, as estimated from the dates of the last HIV antibody seronegative and first seropositive visit. Subsequently, the next 20 top ranked samples from HESN and LESC (also only individuals of European descent and with no CCR5Δ32 alleles) were tested (resulting in 40 HESN and 40 LESC). Absolute counts of circulating CD4+ and CD8+ T cells were available for all subjects. The sample selection procedure employed did not rule out the possibility that some LESC may have been viral RNA+ but seronegative at the visit evaluated. However, transcriptome analysis of cellular RNA in the top ranked 20 HESN and LESC failed to reveal HIV infection (data not shown).

### RNA extraction and cDNA synthesis

Total RNA was extracted from cryopreserved PBMC using the QIAamp RNA Blood Mini Kit (Qiagen, USA) and further purified with the TURBO DNA-Free Kit (Ambion, USA) to remove residual genomic DNA. Purified RNA was then amplified with primers specific to the reference gene ODC1 (forward: 5’-TGGTAATGAAGAGTTTGACTGCCACTT-3’; reverse: 5’CACTGAGACGACAGACTGCTTTGGAA-3’) without prior reverse transcription to confirm the complete removal of genomic DNA; 10 ng genomic DNA was used as a positive control. RNA was then reverse transcribed with a CDKN1A gene specific primer (5’- AGTGCCAGGAAAGACAACTACT-3’) at a final concentration of 100 nM, and SuperScript III Reverse Transcriptase (Invitrogen, USA) according to the manufacturer’s protocol.

### Real time PCR

This assay was performed in triplicate in 25 ul reaction volumes composed of: 1X Gene Expression Master Mix (Applied Biosystems, USA), 1X CDKN1A TaqMan Gene Expression Assay reagents (Applied Biosystems, Hs00355782_m1) and 2.5 ng cDNA. Water and human genomic DNA (500 ng) were used as negative controls (the latter since the probe spans the exon 2/exon 3 junction of the spliced RNA). Reactions were heated to 50°C for 2 minutes, 95°C for 10 minutes, and then 40 cycles of 95°C for 15 seconds and 60°C for 1 minute. The sequence of the CDKN1A clone (Open BioSystems, Image 3355833) was verified and used to construct a standard curve ranging from 3 (lower limit of quantification for the assay) to 3x10^5^ copies. p21 cDNA copy numbers were determined from amplification plots using ABI7500 software v2.0.1 (Applied Biosystems, USA).

### Statistical analysis

Differences in CD4, CD8, CD4:CD8 ratio, and p21 expression data between HESN and LESC groups were tested for statistical significance with the Mann-Whitney U test.

## Results

### Risk levels for HIV infection of HESN and LESC assessed by the number of reported unprotected sex partners

The 20 individuals with the greatest risk exposure (highest *pscore*; highest number of individual sex partners) in the 2.5 years before MACS visit 2 (1984–5) had 80–401 partners during that time period (median = 111), with the next 20 highest risk individuals having between 50–66 partners (median = 53). Twenty individuals that went on to seroconvert within 1 year, but with the least risk exposure, had between 0–2 partners (median = 1), while the next group of 20 had between 2–6 partners (median = 3). Taken together, the top 40 HESN had a median of 73 partners, while the top 40 LESC had a median of 2 partners in the 2.5 years prior to sampling.

We found weak trends between HESN status and lower CD4+ T-cell counts (Mann-Whitney U test, *P* = 0.13; HESN median = 899 cells/uL; LESC median = 1116) (S1 Fig.) and CD8+ T-cell counts (Mann-Whitney U test, *P* = 0.13; HESN median = 570 cells/uL; LESC median = 700) when comparing the top 20 ranked HESN and LESC, but no significant linear correlation between these values and risk of exposure (S2 Fig.). Furthermore, CD4:CD8 ratios (Mann-Whitney U test, *P* = 0.84; HESN median = 1.48; LESC median = 1.31) and CD4 percentages were not significantly different between the risk groups (P = 0.86) (S1 Fig.).

### No consistently observed differences between p21 expression in very high risk HESN and very low risk LESC

**Fig 1:**
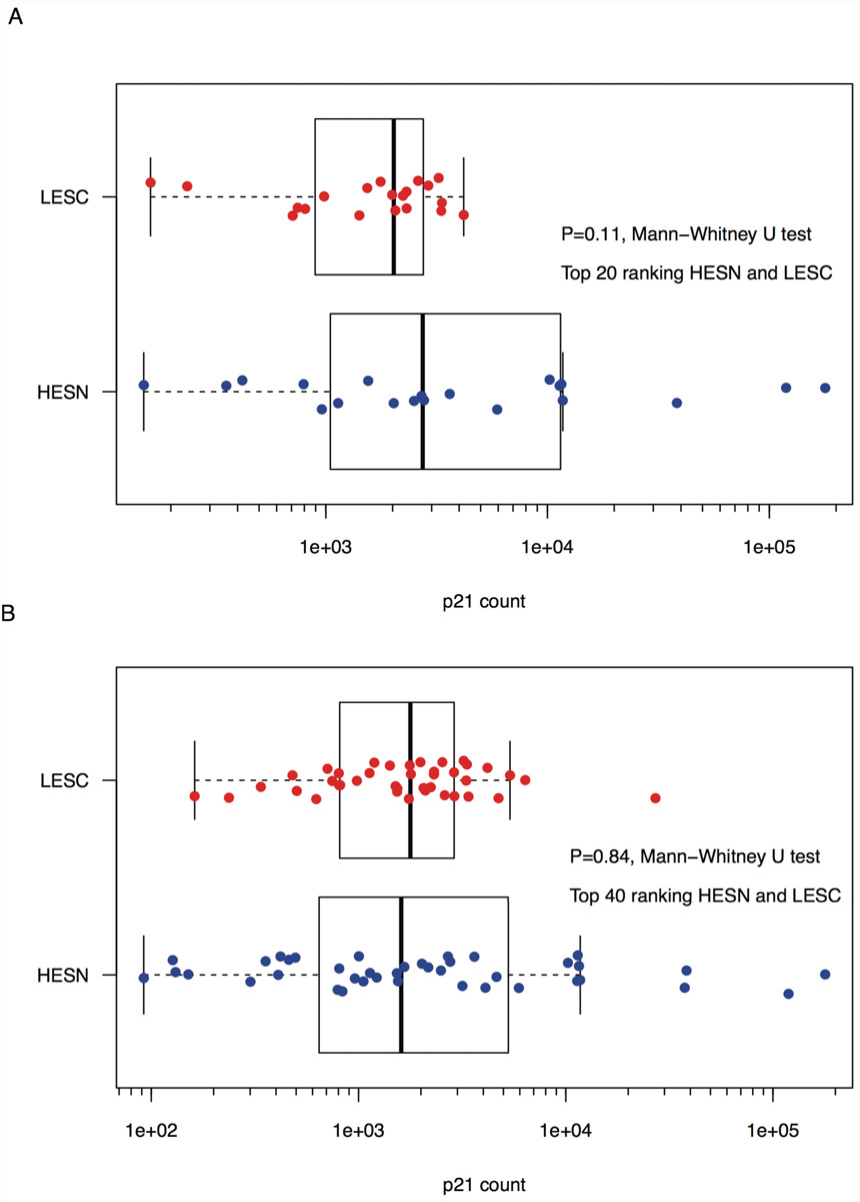
p21 expression in HESN and LESC. Shown are comparisons of p21 expression between: A) the top 20 ranking HIV-uninfected individuals with the greatest risk exposure (HESN) and the top 20 ranking HIV-infected individuals with the lowest risk exposure (LESC) (sampled prior to HIV infection); and B) the top 40 ranking HESN and LESC. All individuals were of European ancestry and none possessed a CCR5Δ32 deletion. p21 count refers to the number of p21 transcripts per ng of cDNA derived from total PBMC RNA.

We conducted 3 experiments to evaluate p21 RNA expression; within each experiment all samples were evaluated in triplicate. In the first experiment, we observed a trend for higher median p21 RNA levels in the top 20 ranked HESN compared to the top ranked 20 LESC (Fig. 1A) (Mann-Whitney U test, *P* = 0.11). Median levels of expression in HESN were 2,734 copies/ng cDNA (range 151 to 178,700) compared to 2,024 in LESC (range 162 to 4,196). However, in a second experiment using the same RNA preparation, no difference was observed between the 2 groups (P = 0.80) (S3 Fig.).

Given the possible trend toward higher p21 RNA expression in the most extreme HESN relative to the most extreme LESC, we assessed more individuals, *i*.*e*., the 20 HESN with the next highest exposure risks and the 20 LESC with the next lowest exposure risks. This analysis found no significant difference between the two groups of 40 individuals in p21 RNA expression (Mann-Whitney U test, *P* = 0.84) (Fig. 1B). The median level of expression for the top 40 ranked HESN was 1,607 copies/ng cDNA (range 92 to 178,700) compared to 1,779 in LESC (range 162 to 27,130). A repeat of this experiment again showed no difference between the two groups of 40 (P = 0.47) (S3 Fig.). Despite the contrasting statistical inferences from the first two experiments, overall, the p21 copy numbers had very high concordance between the initial and replicate experiments (R^2^ = 0.90, *P* = 2x10^-16^ for all 80 p21 measurements across both risk groups) (S4 Fig.).

**Fig 2:**
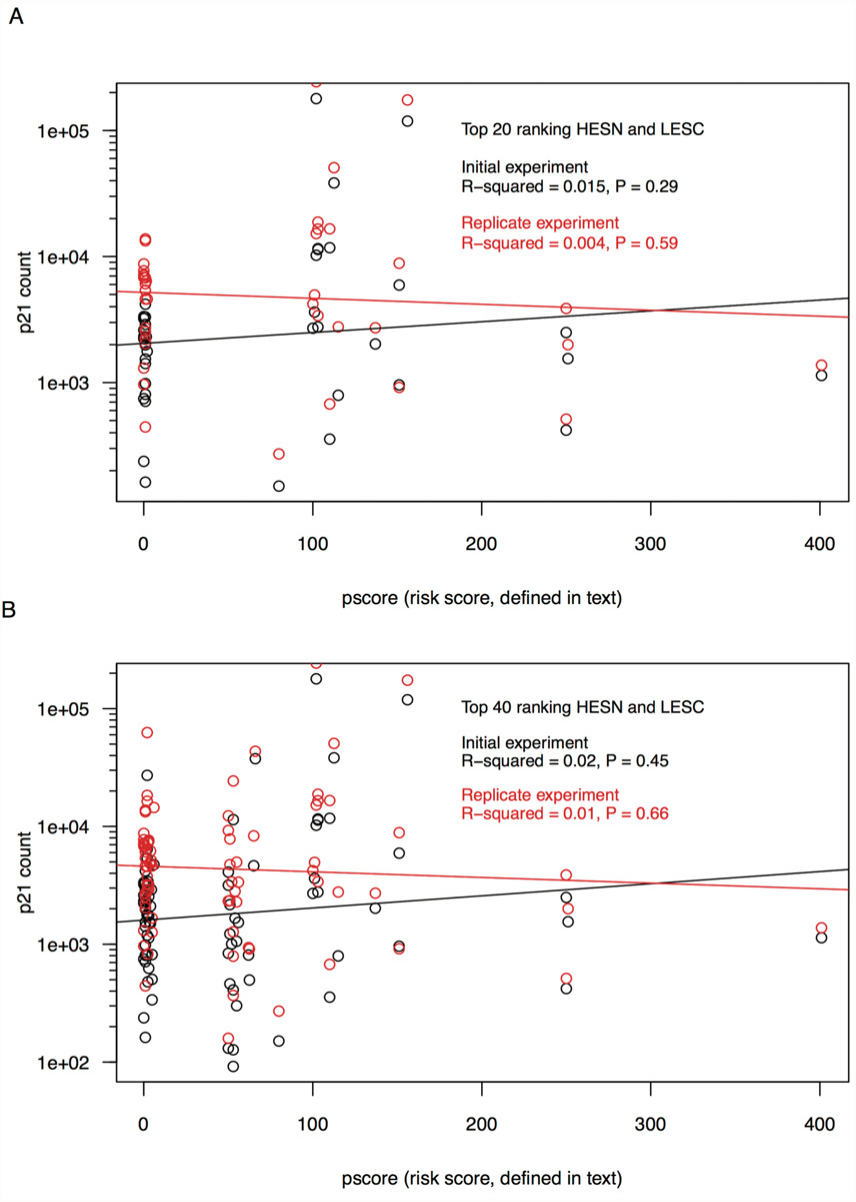
Linear regression comparisons of p21 expression in HESN and LESC. A) Linear regression of log10-transformed p21 count (number of p21 transcripts per ng of cDNA derived from total PBMC RNA) on individual *pscores* (the partner score, determined by the number of self-reported different sexual partners and the proportion of those partners with whom the individual had unprotected anal-receptive intercourse in the previous 2.5 years) for the top 20 ranking HESN and LESC. Shown are initial (black) and replicate (red) experiments. All individuals were of Caucasian ancestry and none possessed the CCR5Δ32 deletion. B) Plot of linear regression of p21 expression data on individual *pscores* for the top 40 ranking HESN and LESC, with initial (black) and replicate (red) experiments shown.

To determine whether there was a relationship between p21 expression and *pscore* considered as a continuous variable, p21 expression levels from the top 40 ranking HESN and the top 40 ranking LESC were compared against individual *pscores* with uncorrected ordinary least squares regression. There were essentially flat and non-significant linear relationships between log10-transformed p21 expression and *pscore* at the individual level, for both the initial and replicate experiments (R^2^ = 0.015, *P* = 0.29, R^2^ = 0.004, *P* = 0.59, respectively) (Fig. 2A). Regression analysis of only the top 20 ranking HESN and top 20 ranking LESC also showed no significant relationship, for both the initial and replicate experiments (R^2^ = 0.02, *P* = 0.45, R^2^ = 0.01, *P* = 0.66, respectively) (Fig. 2B).

## Discussion

HIV-1 infection is an inefficient and complex process that can be influenced by many factors, including the route and magnitude of virus exposure as well as innate host factors, only one of which, the Δ32 mutation in the HIV co-receptor CCR5, has been consistently identified as affecting risk of infection *in vivo* [5]. Critical examination of extreme risk groups, as described here, is needed to determine additional host correlates of HIV-1 infection. The results of the present study suggest that p21 levels play little if any role in protection from HIV infection, despite some prior data indicating that high levels of p21 expression is associated with HIV controller status [30], resistance of HIV target cells to infection in cell culture [26,28] and lower HIV expression in cell culture [29]. However, the limitations of the current study should be noted: it had relatively small sample sizes and no direct measurement of HIV exposure, and unfractionated PBMC were evaluated; it is possible that p21 expression may be different in pure CD4+ T cell or activated CD4+ T cell populations, where it would be expected to have the greatest impact on HIV-1 infection. We could not test this possibility because of limited cell availability. Thus, although p21 is a hypothetically strong candidate, and perhaps a minor contributor, to natural protection from HIV-1 infection in HESN, a broader search is required to identify additional major host factors responsible for this protection.

## Supporting Information

S1 Fig Distributions of CD4+ T cell counts in HESN and LESC. Shown are comparisons of CD4 cells/uL (panel A) and CD4:CD8 ratio (panel B) between the top 20 ranking seronegative individuals with the greatest risk exposure (HESN) and the top 20 ranking HIV-infected individuals with the lowest risk exposure (LESC) (sampled prior to HIV infection).
(TIF)

S2 Fig Linear regression of CD4+ T cell counts versus *pscore*. No significant linear correlation between CD4+ T cells/uL values and risk of exposure for the top 20 ranked HESN and LESC.
(TIF)

S3 Fig Distributions of p21 expression in HESN and LESC from replicate experiments. Shown here are comparisons of p21 expression, produced from a replicate experiment, between: A) the top 20 ranking seronegative individuals with the greatest risk exposure (HESN) and the top 20 ranking seroconverting individuals with the lowest risk exposure (LESC); and B) the top 40 ranking HESN and LESC.
(TIF)

S4 Fig Comparison of p21 expression levels estimated in a replicate experiment. Shown here are comparisons of p21 expression produced from the initial and the replicate experiments for all the top 40 ranking HESN and LESC (for a comparison of 80 p21 measurements in total).
(TIF)
