# Supplementary figures and images for: p21WAF1/CIP1 RNA Expression in Highly HIV-1 Exposed, Uninfected Individuals
Source: PLoS One. 2015 Mar 6;10(3):e0119218. doi: 10.1371/journal.pone.0119218 (PMC4352077; doi:10.1371/journal.pone.0119218)

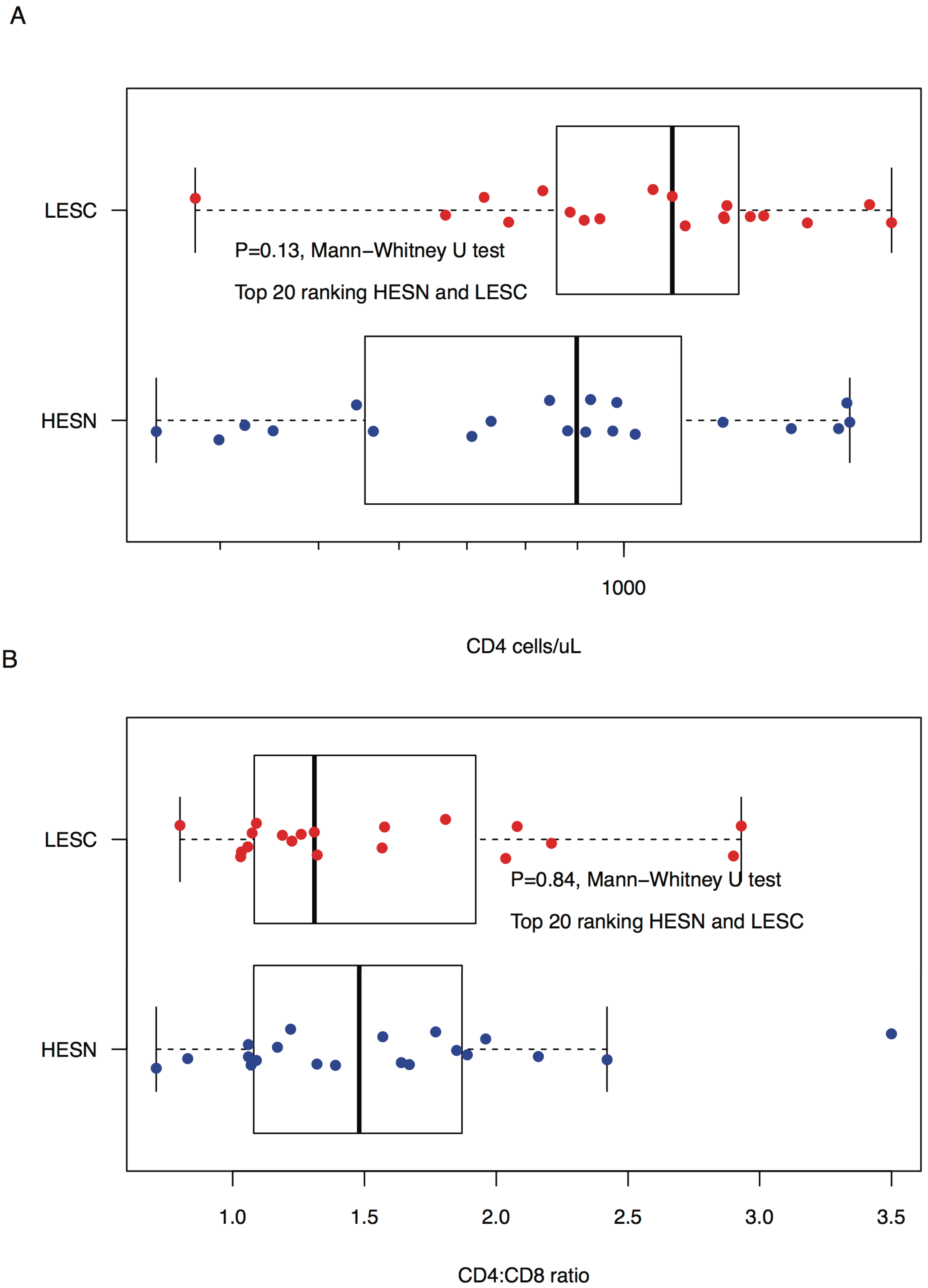

Supplement: S1 Fig — Shown are comparisons of CD4 cells/uL (panel A) and CD4:CD8 ratio (panel B) between the top 20 ranking seronegative individuals with the greatest risk exposure (HESN) and the top 20 ranking HIV-infected individuals with the lowest risk exposure (LESC) (sampled prior to HIV infection). (TIF) [file pone.0119218.s001.tif]

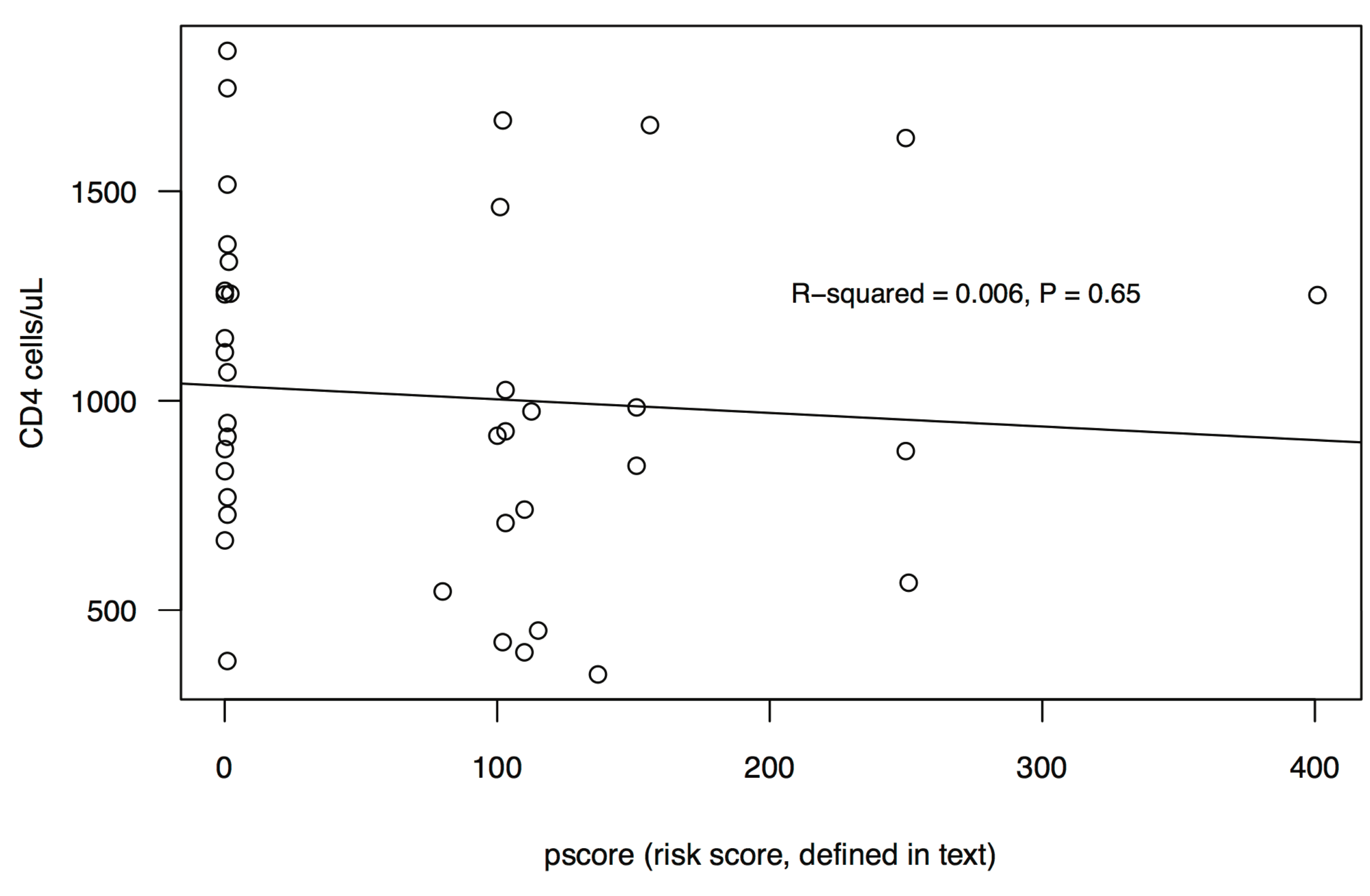

Supplement: S2 Fig — No significant linear correlation between CD4+ T cells/uL values and risk of exposure for the top 20 ranked HESN and LESC. (TIF) [file pone.0119218.s002.tif]

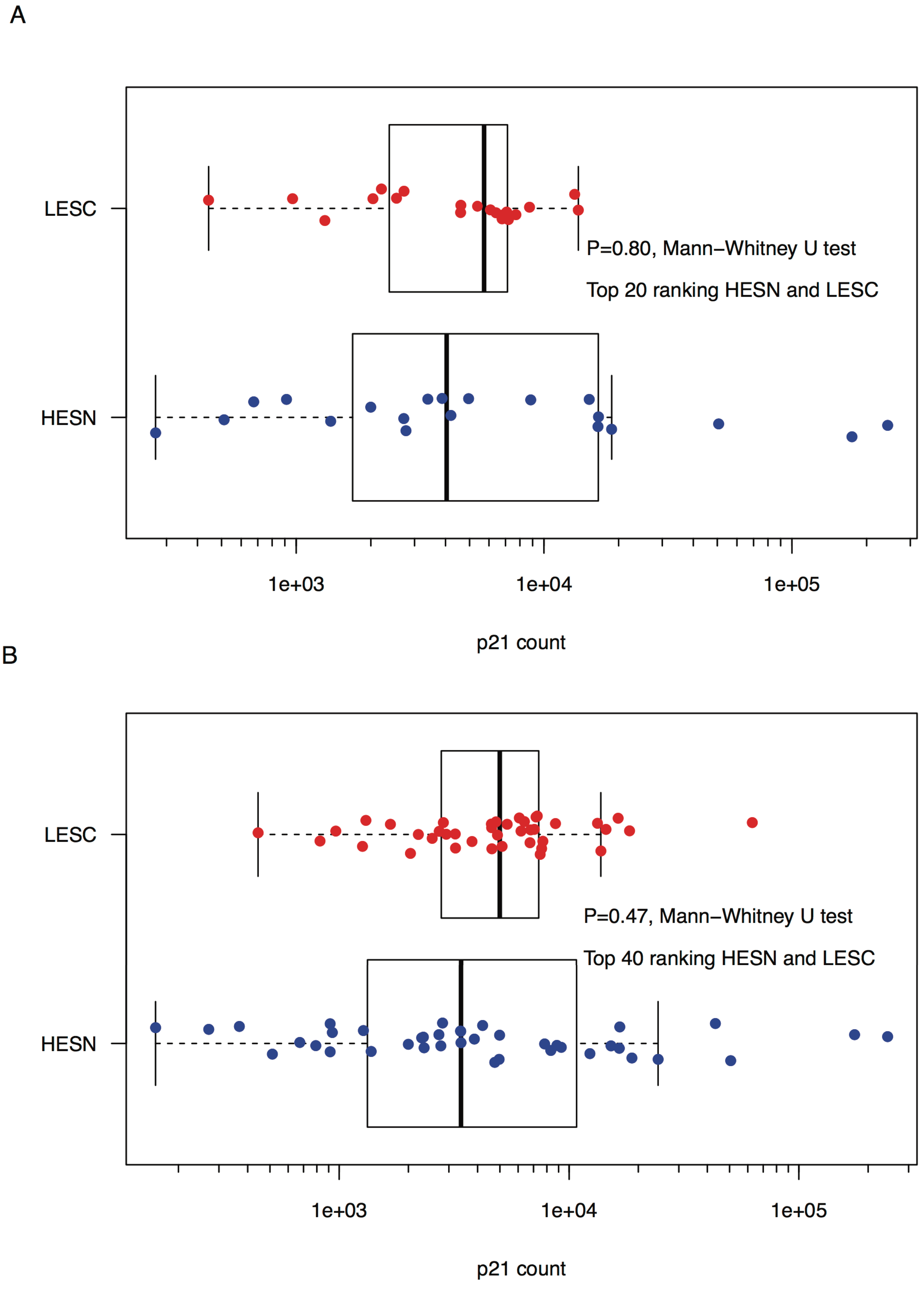

Supplement: S3 Fig — Shown here are comparisons of p21 expression, produced from a replicate experiment, between: A) the top 20 ranking seronegative individuals with the greatest risk exposure (HESN) and the top 20 ranking seroconverting individuals with the lowest risk exposure (LESC); and B) the top 40 ranking HESN and LESC. (TIF) [file pone.0119218.s003.tif]

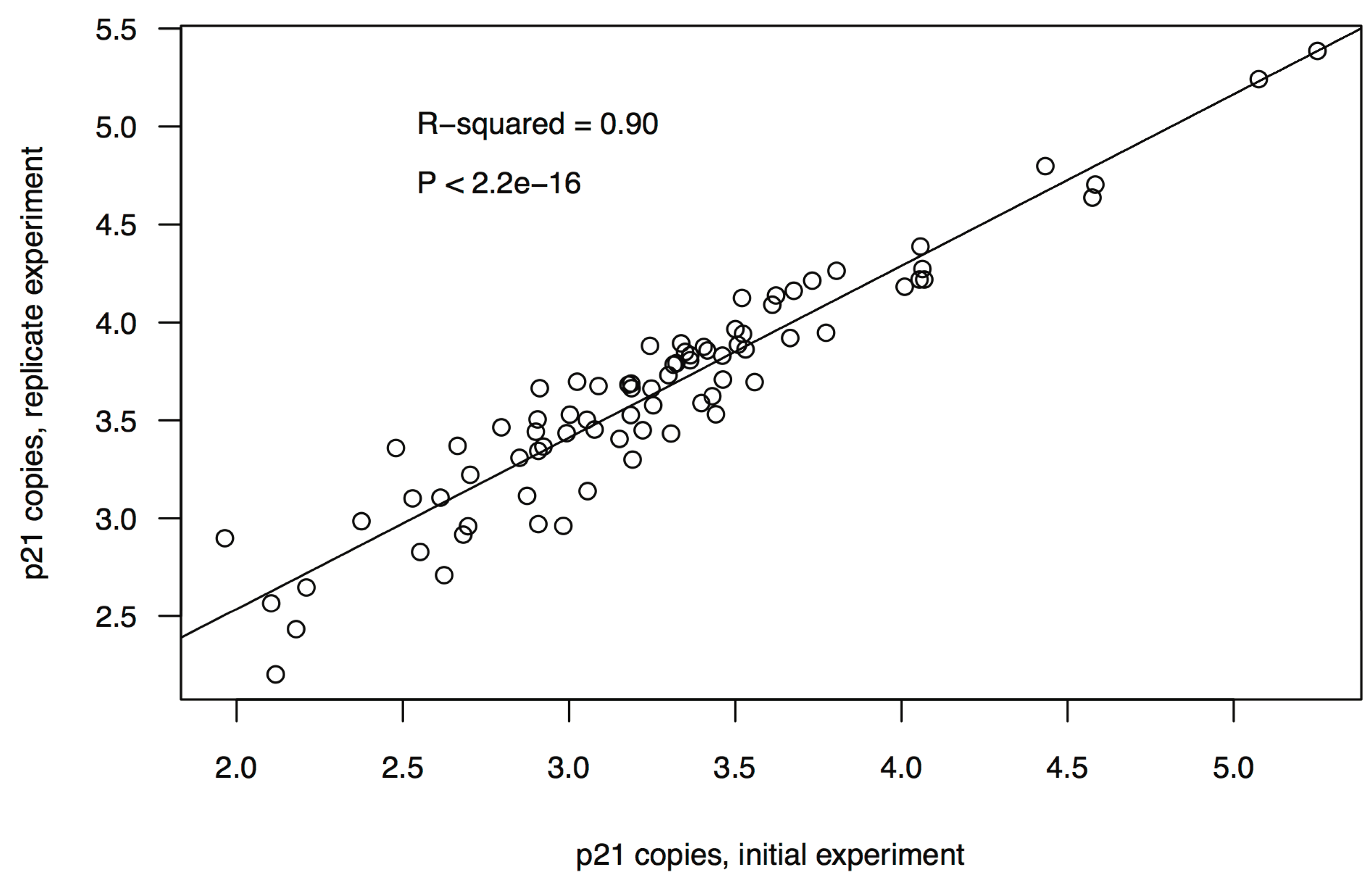

Supplement: S4 Fig — Shown here are comparisons of p21 expression produced from the initial and the replicate experiments for all the top 40 ranking HESN and LESC (for a comparison of 80 p21 measurements in total). (TIF) [file pone.0119218.s004.tif]
